# The Effect of Chain Length and Conformation on the Nucleation of Glycine Homopeptides during the Crystallization Process

**DOI:** 10.1021/acs.cgd.2c01229

**Published:** 2023-01-24

**Authors:** Mingxia Guo, Marie J. Jones, Racheal Goh, Vivek Verma, Emily Guinn, Jerry Y. Y. Heng

**Affiliations:** † Department of Chemical Engineering, Imperial College London, South Kensington Campus, LondonSW7 2AZ, U.K.; § Institute for Molecular Science and Engineering, Imperial College London, South Kensington Campus, LondonSW7 2AZ, U.K.; ‡ Synthetic Molecule Design and Development, Lilly Research Laboratories, Eli Lilly and Company, Indianapolis, Indiana46221, United States

## Abstract

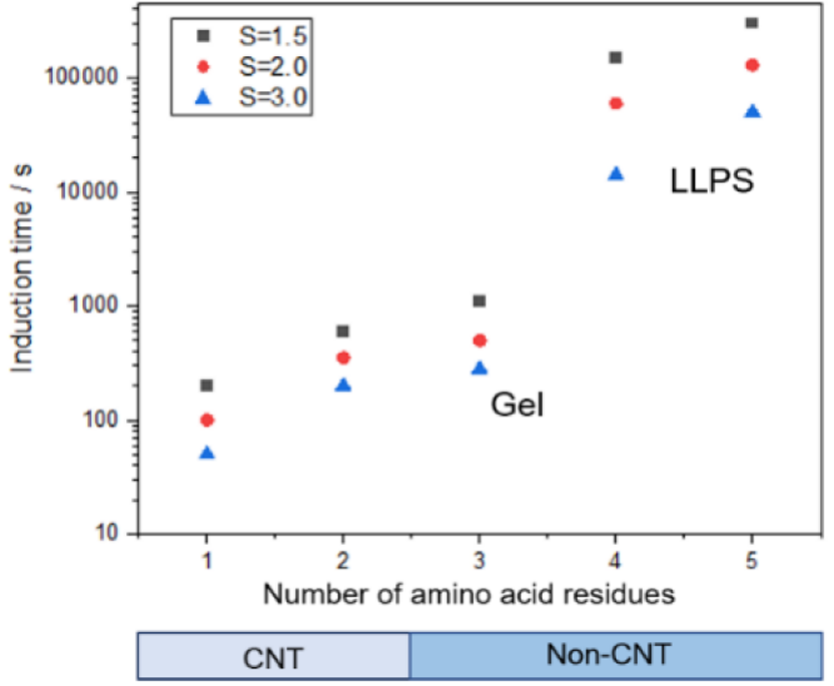

To explore the effect of chain length and conformation on the nucleation of peptides, the primary nucleation induction time of glycine homopeptides in pure water at different supersaturation levels under various temperatures has been determined. Nucleation data suggest that longer chains will prolong the induction time, especially for chains longer than three, where nucleation will occur over several days. In contrast, the nucleation rate increased with an increase in the supersaturation for all homopeptides. Induction time and nucleation difficulty increase at lower temperatures. However, for triglycine, the dihydrate form was produced with an unfolded peptide conformation (pPII) at low temperature. The interfacial energy and activation Gibbs energy of this dihydrate form are both lower than those at high temperature, while the induction time is longer, indicating the classical nucleation theory is not suitable to explain the nucleation phenomenon of triglycine dihydrate. Moreover, gelation and liquid–liquid separation of longer chain glycine homopeptides were observed, which was normally classified to nonclassical nucleation theory. This work provides insight into how the nucleation process evolves with increasing chain length and variable conformation, thereby offering a fundamental understanding of the critical peptide chain length for the classical nucleation theory and complex nucleation process for peptides.

## Abstract 2

The effects of chain length and conformation on the nucleation of glycine homopeptides have been investigated here. The induction time increases with the peptide chain length, and nucleation parameters of triglycine dihydrate with pPII conformation indicate that classical nucleation theory (CNT) is not suitable to explain its nucleation phenomenon. Moreover, gelation and liquid−liquid separation of longer chain glycine homopeptides were observed.

## Introduction

Nucleation is the first and essential step in the process of crystallization, where a new thermodynamic phase forms with lower free energy.^1^ Nucleation kinetics plays a decisive role in the control of polymorphism and crystalline product quality.^2^ The current crystal nucleation research can be explained through classical and nonclassical nucleation theories.^3−6^ Among them, the classical nucleation theory remains the most common theoretical model for the understanding of nucleation, which suggests that concomitant density and order fluctuations cause the formation of clusters, and following that clusters begin to aggregate to form the nucleus.^7,8^ The nucleation process of macromolecule crystallization has been explored by numerous pathways in the past decade.^9,10^ With complex secondary, tertiary, and quaternary structures, classical nucleation theory which can be applied well to explain small molecule nucleation has encountered a formidable obstacle in regard to proteins. Nonclassical nucleation theories, such as the two-step theory and the prenucleation clusters theory, have been proven to explain a specific protein nucleation process.^11−14^ Therefore, these theories put forward questions such as, what is the critical chain length of peptides to distinguish these two different nucleation theories? Is there any relationship between the conformation and the nucleation mechanism?

Peptides are structurally comparable to proteins due to the presence of peptide bonds and amino acid residues, but most of them have a simpler space structure without any tertiary and quaternary structure. Additionally, the short chain length makes it looks like small molecules in molecular size. Therefore, peptides serve as an excellent model for studying the crucial chain length at which classical nucleation occurs. Few studies have been conducted on the nucleation mechanism for peptides, making it imperative to determine the link between small molecule nucleation and protein nucleation.

Glycine is the simplest amino acid with a single hydrogen atom as its side chain, allowing study on the nucleation of glycine homopeptides to be conducted without concerning the effect of side chains. The solubilities of glycine homopeptides (from mono- to pentaglycine) were measured using UV–vis in our previous work.^15^ In another work, triglycine was found to form a dihydrate with unfolded pPII (polyproline II) conformation under 303 K.^16^ Based on these thermodynamic and morphological research studies, the primary nucleation induction time of glycine homopeptides at different supersaturation levels (1.4 to 2.4) and temperatures (278.15 K and 283.15 K) was investigated to determine the effect of peptide chain length and conformation on the nucleation of peptide crystallization. At each condition, 100 experiments have been conducted to capture the statistics of the nucleation process. The data were analyzed using the probability distribution function of the induction time within the framework of classical nucleation theory. Overall, the nucleation parameters (nucleation rate *J*, growth time *t*_g_, interfacial energy γ, critical radius: *r*_C_, number of molecules in critical nucleus *n*_C_, and activation Gibbs energy Δ*G*_C_) of glycine homopeptides have been calculated and compared.

## Theory

### Induction Time *t*_d_

Before a nucleus can be detected by instruments or human vision, it must have reached a certain size or be present in a large number, which means that the time *t*_n_ for a single nucleus to be formed cannot be accurately recorded. The period of time between the moment of a constant supersaturation created and the formation of crystals which can be detected is defined as “induction time” (*t*_d_).^5^ The nuclei have to grow to a detectable size in order to obtain measurements since it is impossible to detect the real induction time (*t*_n_) when the critical nuclei form. Due to this, evaluating “induction time” is the only way. Induction time is larger than *t*_n_ and cannot be regarded as a fundamental characteristic of the system because its values depend on the method used to identify the emergence of a new phase. Examining the values for induction time, however, can help comprehend the mechanisms of new phase formation and growth from critical nuclei into crystals.^17^

The induction time can be expressed as the sum of three terms as follows:
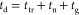
1where *t*_d_ is the measured induction time, *t*_tr_ is the time needed for reaching steady-state nucleation, *t*_n_ is the nucleation time, and *t*_g_ is the growth time required for the critical nucleus to grow to a larger detected size.

By determining the induction time, the nucleation kinetics can be calculated and used to find the thermodynamic and kinetic parameters (such as interfacial energy, critical nucleation free energy, pre-exponential factor, and critical radius and number of molecules in the nucleus) required for efficient crystallization design which will be beneficial for future works in crystallization.

### Determination of Nucleation Rate *J*

Due to the stochastic nature of crystallization, the measured induction times of glycine homopeptides can be approximated to a cumulative probability distribution *P*(*t*). For *N* isolated experiments, the probability *P*(*t*) of observing an induction time between time zero and *t* is defined as follows:
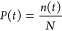
2where *n*(*t*) is the number of trials for which crystals were detected at time *t*.

The experimentally determined cumulative probability distribution for the induction times was found by Jiang and ter Horst to resemble the Poisson Distribution.^18^ They have indeed expressed the probability of finding at least one nucleus at time *t*_*n*_ as the Poisson distribution:
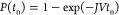
3where *J* is the nucleation rate and *V* is the volume of solution.

Besides, the probability distribution for the detection time can thus be rewritten on the model of the Poisson Distribution as eq 4. The transient period *t*_tr_ can be ignored here since it is unimportant in aqueous solutions of moderate supersaturations and viscosities.^19^
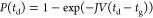
4

### Nucleation Data Derived from Classical Nucleation Theory

The Classical Nucleation Theory was used to determine the thermodynamic factor *B* from which nucleation data can be derived and compared for each glycine homopeptide.

The supersaturation *S* can be expressed as
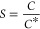
5where *c* is the actual concentration of the solution, c* is the solubility at the specific temperature, and the unit of all data is mole fraction.

The relation between the nucleation rate *J*, the induction time obtained experimentally, and supersaturation *S* could be expressed as the following equations based on different limiting steps:^20−22^
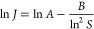
6
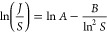
7
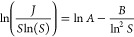
8
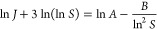
9where *A* and *B* are usually considered to be constants, and the exponent *B*/ln 2 *S* = W/*kT* is the dimensionless nucleation energy barrier for nucleation.

Equations 6 and 7 is for interface-transfer control, eq 8 is for volume-diffusion control, and eq 9 is another interface-transfer control expression according to the assumption that nucleus growth takes place by a surface nucleation mechanism. Equation 9 was plotted for each polyglycine in this work, and the slope of this linear regression provided an estimation of factor *B*, defined as
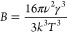
10where ν is the volume of one molecule, γ represents the interfacial energy, *k* is the Boltzmann constant, and *T* is the absolute temperature in Kelvin.

Interfacial energy is the work required to generate a new interface between the supersaturated solution and the solid phase contacting it.^23^ Critical radius *r*_C_ is the critical radius that corresponds to the minimum size at which a particle can survive in a solution without being redissolved.^24^ Number of molecules in critical nucleus *n*_C_ is the number of molecules that must be included in the initial nucleus. Activation Gibbs energy Δ*G*_C_ is the energy barrier that the cluster needs to conquer to form a nucleus during the homogeneous nucleation process.^1^ From the thermodynamic factor *B*, the interfacial energy γ and the following nucleation parameters can be calculated.^22^Interfacial energy
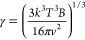
11Critical radius:.
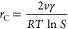
12Number of molecules in the critical nucleus
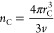
13Activation Gibbs energy
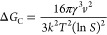
14where ν is the volume of one molecule, *S* is the supersaturation, γ represents the interfacial energy, *k* is the Boltzmann constant, and *T* is the absolute temperature in Kelvin.

## Experimental Section

### The Solid-State Characterization of Glycine Homopeptides

PXRD patterns were collected by a PANalytical X’Pert PRO X-ray diffractometer. Samples were filtrated and pressed into the sample holder. At 40 kV and 40 mA, Cu Kα radiation (1.5405 Å) was used to accomplish the X-ray diffraction experiment. All samples were scanned at a rate of 1 step/s throughout a diffraction angle range of 2 to 50°.

### Induction Time Measurement

The induction times of glycine homopeptides crystallization were determined in water at two different temperatures, 278.15 K and 283.15 K. Solutions with different supersaturation levels (listed in Table S2) were prepared for each glycine homopeptide in test tubes. For glycine and diglycine, 2 mL solutions were made with 2 g of deionized water, whereas a volume of 1.5 mL was preferred for triglycine due to the increased feasibility of nucleation. The tubes were equipped with a small magnetic stir bar and meticulously sealed with rubber lids wrapped with parafilm both inside and outside the cap. The samples were placed into the hot thermostat bath maintained at 333.15 K, well above the supersaturation temperature of glycine homopeptides. The solutions were stirred at 500 rpm in the water bath until all the glycine homopeptides had fully dissolved (Figure S1). The clear tubes were then immersed in a cold thermostat bath held at a low constant nucleation temperature (278.15 K and 283.15 K). During the experiment, the solutions of different supersaturations for each peptide were tested in parallel and continuously magnetically stirred at 250 rpm. The induction time was recorded as the time of the first observation of the solution becoming cloudy. Once the solutions had nucleated, the tubes were transferred back to the water bath held at a temperature above the supersaturation temperature, to dissolve before repeating the nucleation experiment cycle. Induction time data were obtained for 100 times for each glycine homopeptide and each supersaturation level to capture the stochastic nature of nucleation. After all the induction time measurements were performed, the solutions were filtered, and the powders were tested using PXRD to determine the morphology of the crystallized glycine homopeptides to make sure there was no polymorphism transformation during the nucleation process.

## Results and Discussion

### The Solid-State Characterization of Glycine Homopeptides

**Figure 1:**
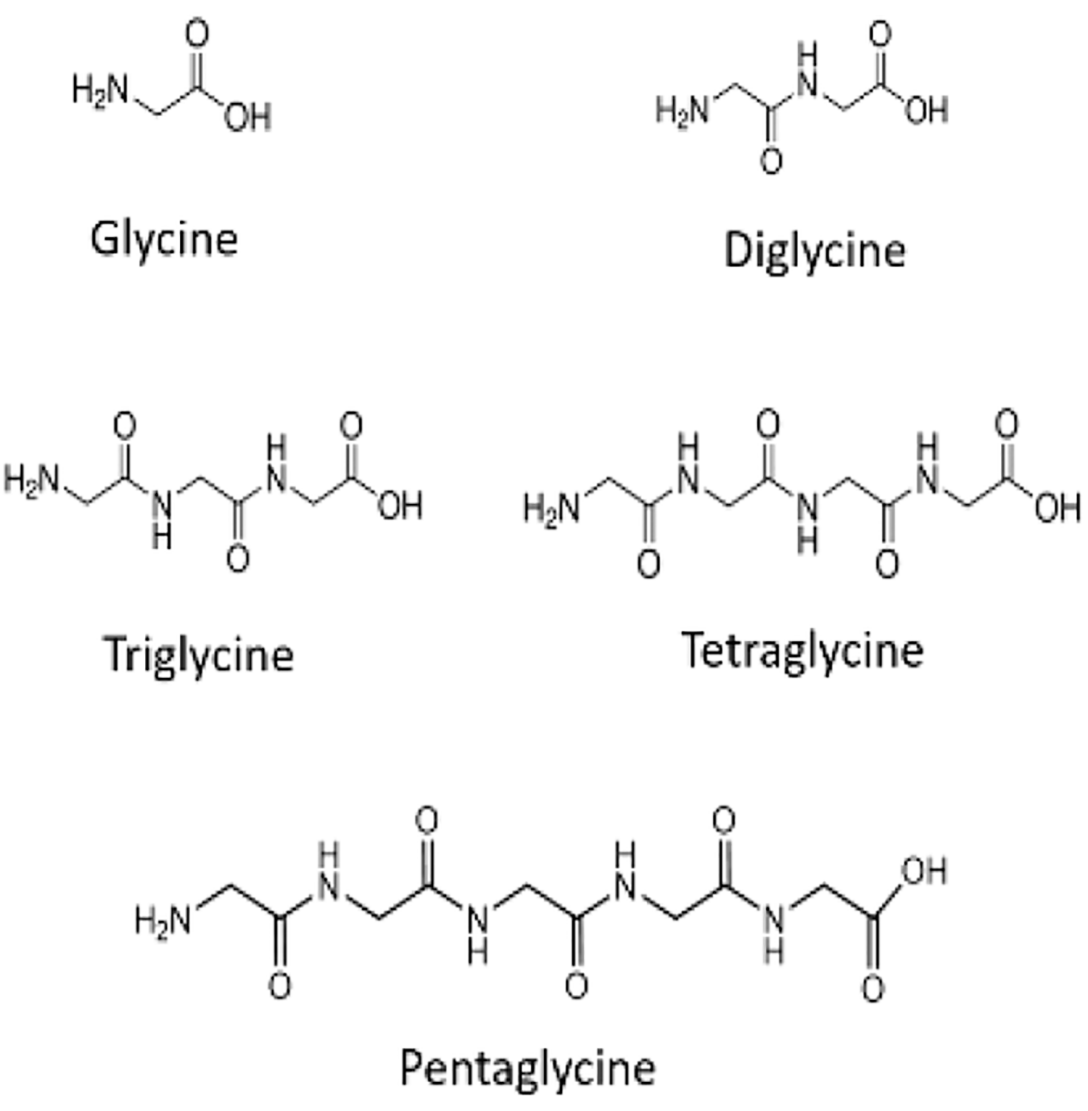
Chemical structures of glycine homopeptides.

Glycine homopeptides (glycine (α form), diglycine (α form), triglycine (β form), tetraglycine, pentaglycine, and hexaglycine) were supplied by Sigma-Aldrich Company Ltd. (Figure 1, Table S1) and were used as received. Deionized water was produced in the laboratory.

The PXRD patterns of glycine homopeptides used in this work have been tested before and after the induction time measurement to make sure that there was no phase transition. As the examples show in Figure S2, all the samples kept the same XRD pattern, except triglycine. The new XRD patterns of triglycine are a result of the dihydrate morphology as reported previously.^16^ The dihydrate form of triglycine can be obtained when the temperature is lower than 303.15 K.

### The Nucleation Parameters of Glycine Homopeptides

**Figure 2:**
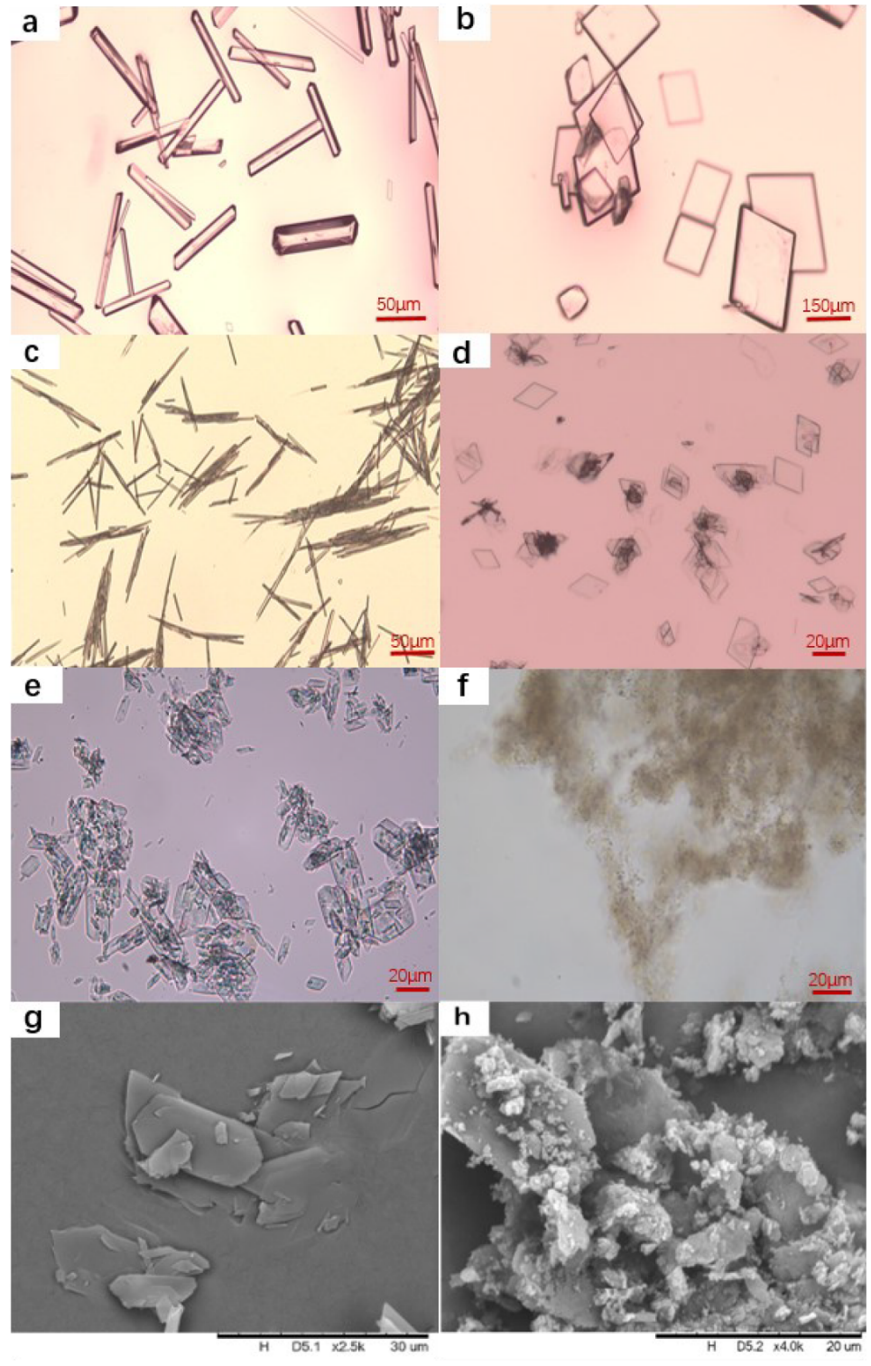
Microscope images of glycine homopeptides (a) glycine, (b) diglycine, (c) triglycine dihydrate, (d) tetraglycine, (e) pentaglycine, (f) hexaglycine, and SEM images of pentaglycine (g) and hexaglycine (h).

The induction times of glycine, diglycine, triglycine, tetraglycine, pentaglycine, and hexaglycine were measured under 278.15 K at different supersaturation levels (1.5, 2, 3) in 2 mL of water. The images and SEM micrographs of the produced crystals are presented in Figure 2. The crystals of glycine homopeptides are all regularly shaped—rodlike for glycine, needle for triglycine dihydrate, plate for di, tetra-, penta-, and hexaglycine. The solution of pentaglycine and hexaglycine became cloudy after several days first until small crystals came out, and the induction time was longer than 1 week.

**Figure 3:**
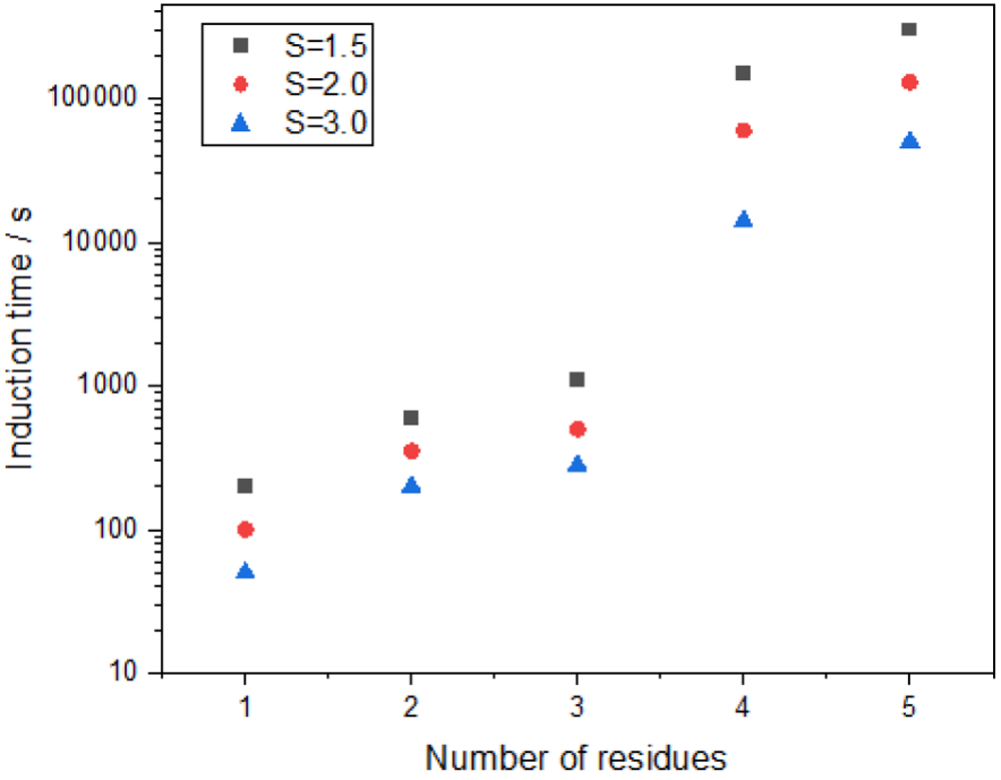
Induction time of glycine homopeptides under different supersaturation levels at 278.15 K.

The experimental results present that the induction time increased with the increasing number of amino acid residues (Figure 3). Glycine has the shortest induction time, whereas pentaglycine has the longest induction time. Moreover, when the number of amino acids in glycine homopeptides exceeds three, the induction time increases by an order of magnitude, which also indicates that when the chain length is long enough, there is no linear relationship between the number of peptide bonds and the nucleation rate. Furthermore, the induction time decreased with the increase of the supersaturation level *S*.

**Figure 4:**
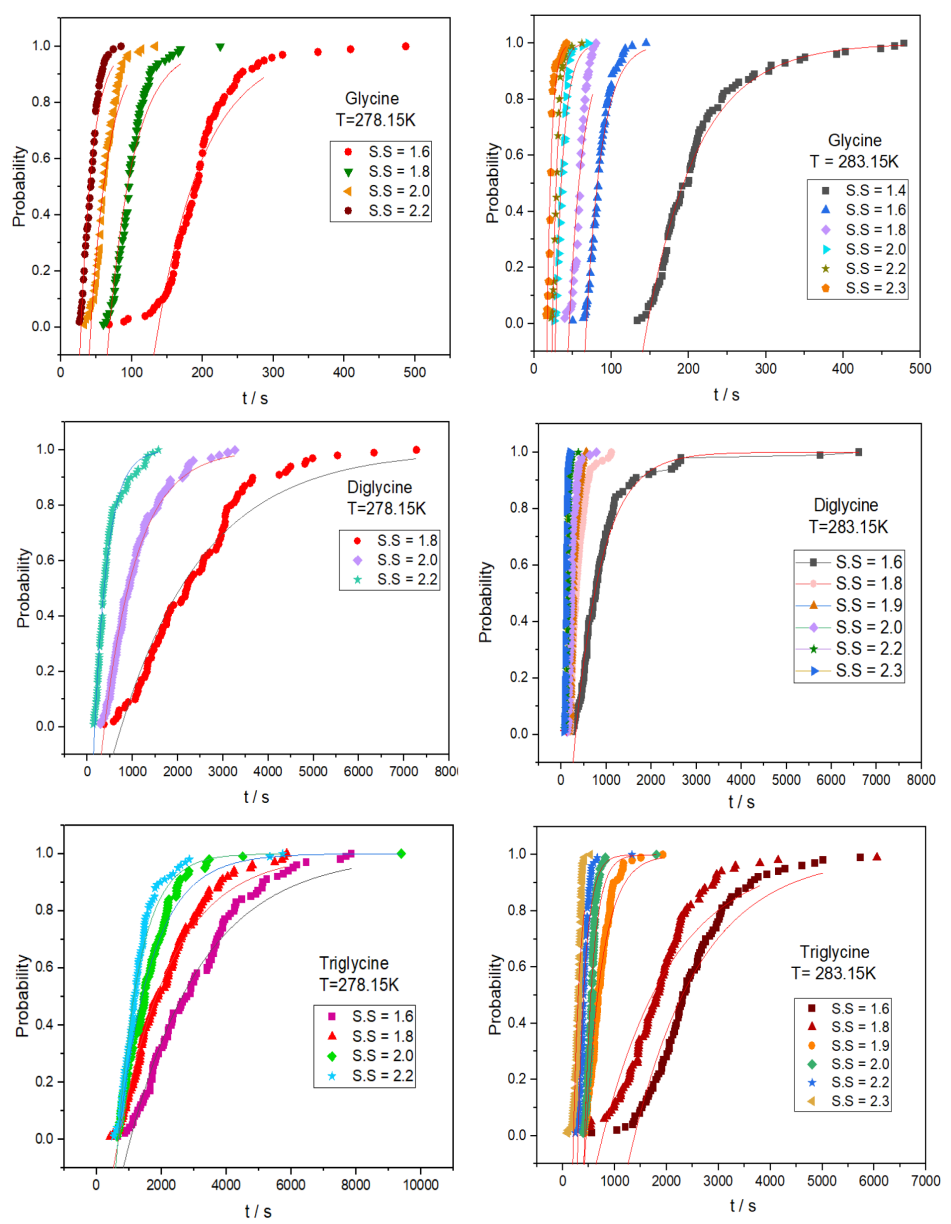
Induction time of glycine homopeptides under different supersaturation levels at 278.15 K and 283.15 K (the Poisson distribution is represented by solid lines with a good fit).

In order to explore the effect of chain length and conformation on the nucleation process, the nucleation parameters of glycine, diglycine and triglycine dihydrate were calculated based on the stochastic nature of nucleation and were compared with each other. Figure 4 and Figure S4 present the probability distribution of the induction times for glycine, diglycine, and triglycine at 278.15 K and 283.15 K. The exponential based Poisson function (eq 3) was utilized to correlate the experimental data, of which the outcome is provided in Table S3. For various rate-limiting steps in the nucleation process, eqs 6–9 can be used to characterize the relationship between the nucleation rate and the supersaturation. The goodness of fit of these four functions was evaluated in order to identify which one best matched the experimental data, as shown in Figure S3. The best fitting findings (the lowest *R*^2^ value) are provided by eq 9, indicating that the nucleation rate for glycine homopeptides in water is regulated by the interface transfer condition, and nucleus growth occurs via a surface nucleation process.

**Figure 5:**
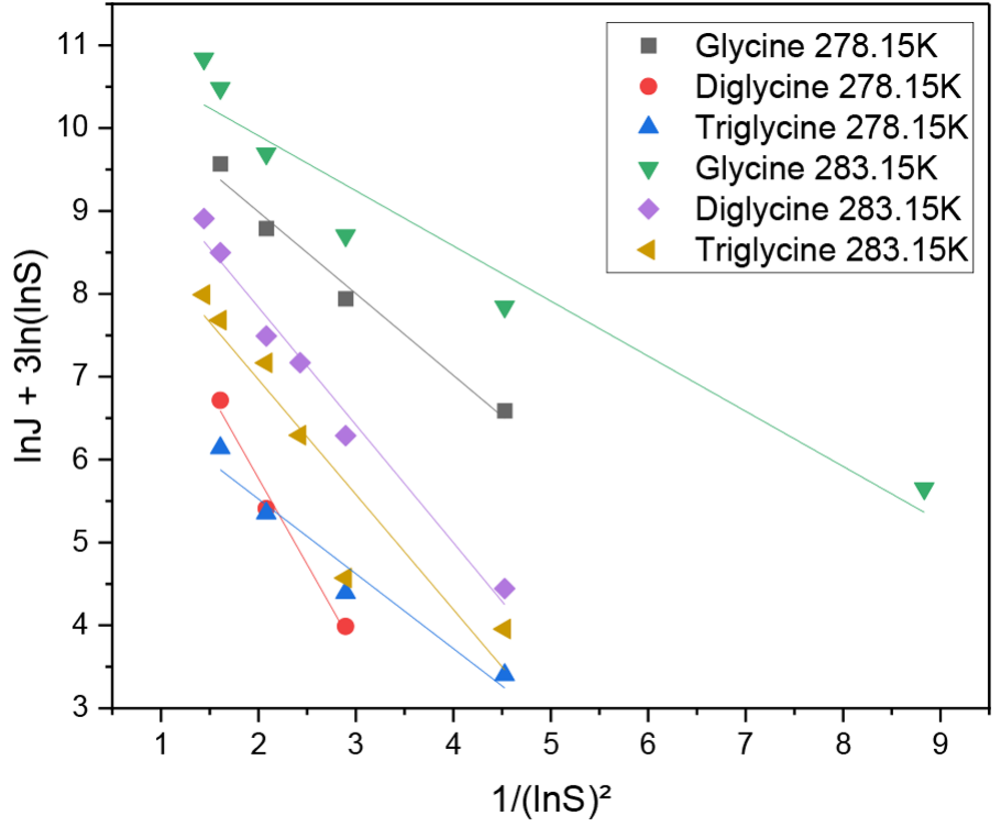
Comparison of the relationships between the nucleation rate and supersaturation level of glycine homopeptides under different temperatures.

The relationship between 1/(ln *S*)^2^ and ln *J* + 3 ln(ln *S*) of glycine homopeptides in eq 9 are shown in Figure 5. From the intercept ln *A* and the slope B of each linear line, the pre-exponential factor *A* and the interfacial energy γ can be determined, respectively. Under same temperature, the steeper the slope, the larger the *B*, and the higher value of υ^2^γ^3^. The activation Gibbs energy Δ*G*_C_ calculated using eq 14 became higher at the same supersaturation level, making the induction time longer. From the interfacial energy, the size of the critical nucleus *r*_C_, the number of molecules *n*_C_ in the critical nucleus, and the free-energy barrier to nucleationΔ*G*_C_ can be calculated by eqs 11–14. The values are listed in Table S4 and Table S5.

According to the results obtained in Tables S4 and S5, as the supersaturation level increases, the Gibbs free energy, critical radius, and number of molecules in one nucleus decrease. This relationship was seen across the different results of the same peptide in both temperatures of different chain lengths. This is potentially because the amount of energy required to form the earliest nucleus of critical size is lower at higher supersaturation levels, and hence, the induction time would be shorter.

**Figure 6:**
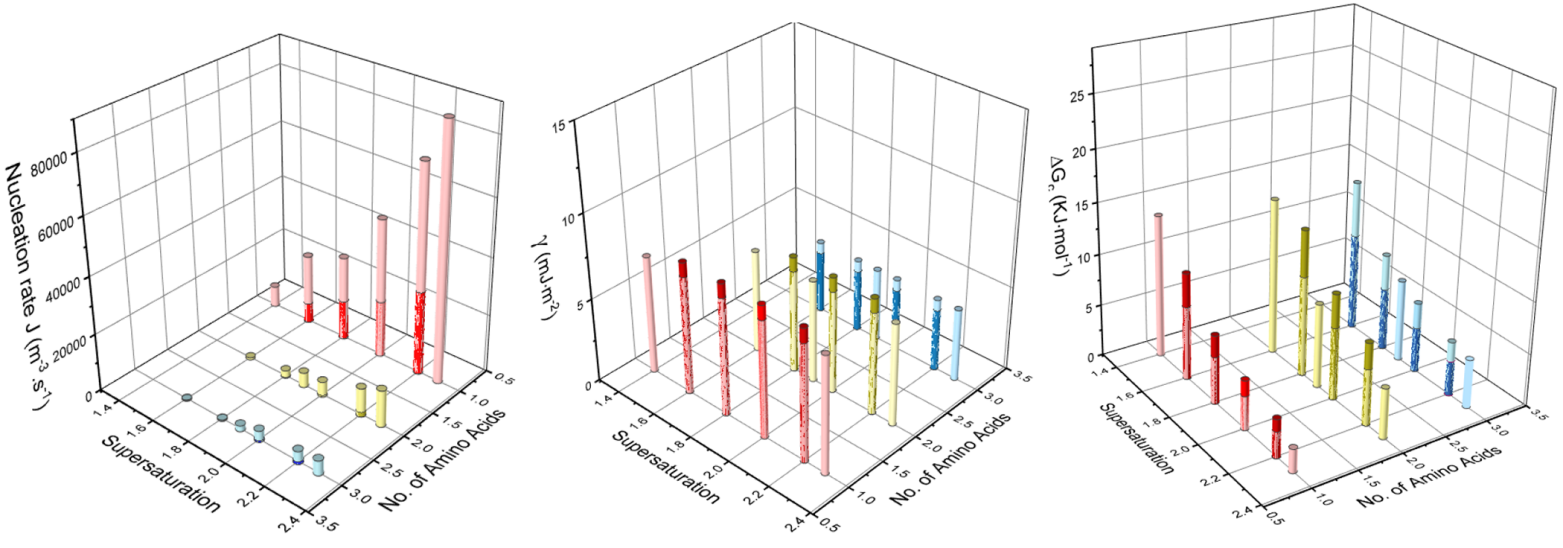
Relationship between nucleation rate *J*, interfacial energy γ, activation energy **Δ***G*_C_ supersaturation levels, and the number of glycine residues. The darker color represents the results at 278.15 K, whereas the lighter color means the results at 283.15 K.

The comparison of nucleation parameters between different peptides is shown in Figure 6. Crystals nucleate more rapidly with a shorter chain length; thus, glycine, which contains only one amino acid, nucleates the quickest and has the highest nucleation rate *J*, while triglycine dihydrate, which has three amino acid residues, nucleates the slowest and has the lowest nucleation rate *J*. The nucleation rate at 283.15 K is greater than that at 278.15 K, which could be because the molecules are more active at a higher temperature, resulting in a faster rate of surface integration and hence a faster nucleation rate.

The kinetics of the new phase transformation (from liquid to solid) process is determined by the surface free energy of the emerging phase boundary, and a greater difficulty in nucleation equals a higher solid–liquid interfacial energy.^13,23^ The order of the interfacial energy of glycine homopeptides in water under the same temperature is as follows: glycine > diglycine > triglycine dihydrate. Triglycine dihydrate is supposed to have the highest γ as an increase in the number of amino acid residues, resulting in an increase in the difficulty of nucleation, but results do not align. From the definition of interfacial energy, it is proportional to thermodynamic factor *B* and inversely proportional to the volume of one peptide molecule. The interfacial energy of various peptides at the same temperature is incomparable because of the different molecular volumes. Since the order of molecular volume is triglycine dihydrate > diglycine > glycine, the order of *B* is diglycine > glycine > triglycine dihydrate at 278.15 K, and triglycine dihydrate > diglycine > glycine at 283.15 K, so triglycine dihydrate can adopt the lowest interfacial energy compared with glycine and diglycine. The influence of temperature on the interfacial energy, on the other hand, can be investigated. When the temperature is increased, all the interfacial energies become lower, except for triglycine dihydrate. However, the induction time of triglycine dihydrate is shorter than that at a lower temperature, which means the interfacial energy of triglycine dihydrate cannot be used as the only evidence of whether the nucleation rate is high or not.

From Figure 6, for all peptides and temperatures, as supersaturation increases, Δ*G*_C_ decreases, indicating that higher driving force accelerates nucleation. When investigating the effect of chain length on activation energy, the energy at the same supersaturation level and temperature was compared. When the temperature is 283.15 K, triglycine dihydrate has the largest Δ*G*_C_ and longest induction time, which aligns with the trend of activation energy. When the temperature is lower as 278.15 K, triglycine has the lowest activation energy compared with mono- and diglycine, which is contradictory with its longest induction time. For mono- and diglycine, Δ*G*_C_ decreases when the temperature is higher, while for triglycine dihydrate, the Δ*G*_C_ increases when the temperature is higher. However, the induction time of triglycine dihydrate is shorter at the higher temperature. Based on our previous research, triglycine will form dihydrate at temperatures below 303 K with the unfolded pPII conformation, while it will convert to anhydrate at higher temperatures with the almost fully extended conformation.^16^ The Ramachandra plot reveals that both the pPII and β-sheet conformations have a strong preference over other alternative conformations in solution at 278 K. The predominance of pPII conformation diminishes as the temperature increases until the conformation is converted to a β-sheet in anhydrate form. For triglycine dihydrate, the unfolded conformation with extra hydrogen bonds formed at a lower temperature, which is not stable with the temperature increasing, causing a higher calculated Δ*G*_C_. In summary, the flexible conformation at varying temperatures rendered the traditional nucleation theory inapplicable to triglycine dihydrate.

**Figure 7:**
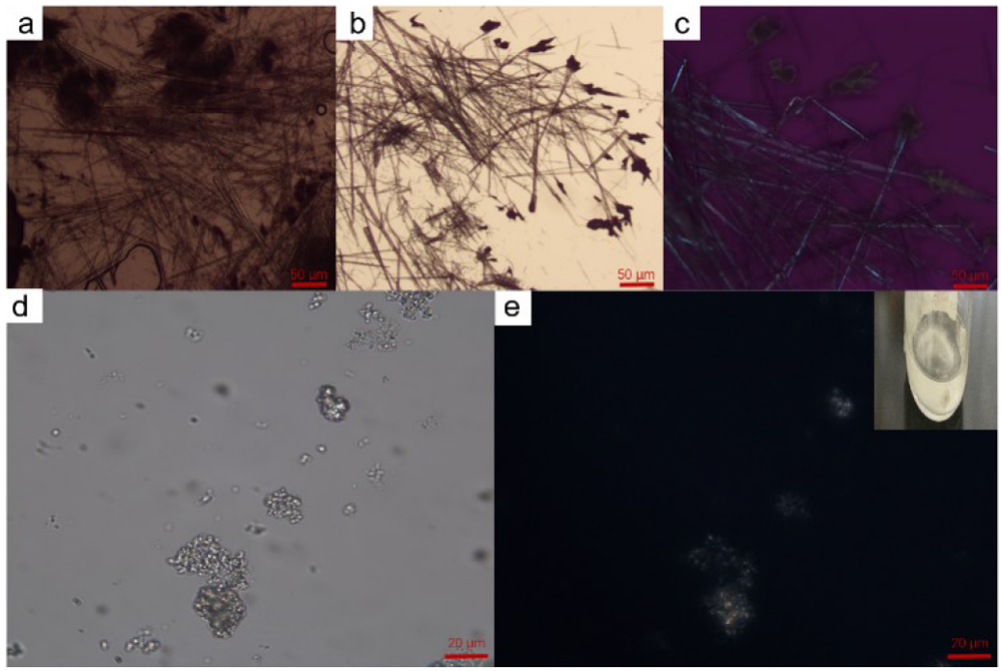
Gelation phenomenon of triglycine dihydrate nucleation captured by an optical microscope (a, b) and polarizing microscope (c) and liquid–liquid phase separation of hexaglycine nucleation captured by an optical microscope (d) and polarizing microscope (e).

Apart from the classical nucleation theory, other nonclassical nucleation models have been developed over the last two decades.^11,26−30^ For the glycine homopeptides, the liquid–liquid phase separation was also observed for hexaglycine before the nucleation, which can be seen in Figure 7d,e. The solution became blurry first, and there were some gel-like particles that will not settle for a long time, similar to the liquid–liquid separation process observed in the literatures.^31,32^

For triglycine, gelation appeared during the crystallization of triglycine dihydrate (Figure 7a–c). This discovery raises a question whether there is any relationship between gelation and liquid–liquid separation during the peptide crystallization. However, because the solubility of hexaglycine is very low, the gelation is difficult to observe. This observation can be further explored to study the nucleation mechanism for longer chain peptides.

## Conclusion

In summary, the nucleation of glycine homopeptides was investigated in this work to explore the effect of chain length and conformation on the nucleation mechanism of short chain homopeptides. The induction time increases with the peptide chain length, which even exhibits an exponential increase when the number of glycine residues exceeds three. For the glycine, diglycine and triglycine, the nucleation parameters (nucleation rate *J*, growth time *t*_g_, interfacial energy γ, critical radius *r*_C_, number of molecules in critical nucleus *n*_C_ and activation Gibbs energy Δ*G*_C_) were calculated. With increasing supersaturation, activation energy, critical radius, number of molecules and growth time decrease, while the nucleation rate increases for all homopeptides. Lower temperature makes the nucleation more difficult and the induction time longer. When the temperature is lower, the interfacial energy γ and Gibbs energy Δ*G*_C_ are higher for glycine and diglycine. However, for triglycine, the dihydrate with unfolded conformation was formed during the nucleation process, and the values of interfacial energy and Gibbs energy at low temperature are both lower than those at high temperature, which means the classical nucleation theory is not suitable to explain the nucleation phenomenon of triglycine dihydrate. Moreover, the gelation phenomenon of triglycine dihydrate was found during the nucleation process, as well as the liquid–liquid separation of hexaglycine. For glycine homopeptides, nonclassical nucleation theory provides a better explanation when the chain length is longer than three with more flexible conformation in solution. This work gives a better understanding of the nucleation mechanism for different chain length peptides.

## Supporting Information Available

Details of the experimental setup, XRD patterns, induction time, the calculated nucleation kinetics parameters, Tables S1–S4, and Figures S1–S4 (PDF)
