# Supplementary material for: The Effect of Chain Length and Conformation on the Nucleation of Glycine Homopeptides during the Crystallization Process
Source: Cryst Growth Des. 2023 Jan 24;23(3):1668–75. doi: 10.1021/acs.cgd.2c01229 (PMC9983003; doi:10.1021/acs.cgd.2c01229)
Supplement: Supplementary file 1 — cg2c01229_si_001.pdf [file cg2c01229_si_001.pdf]

Supporting Information

The effect of chain length and conformation on the nucleation of glycine homopeptides during the crystallisation process

Mingxia Guo[a], Marie J. Jones[a], Racheal Goh[a] Vivek Verma[a], Emily Guinn[b] and Jerry Y.Y. Heng[ab]*

[a]*Department of Chemical Engineering,* [c]*Institute for Molecular Science and Engineering, Imperial College London, South Kensington Campus, London SW7 2AZ, UK*
[b]*Synthetic Molecule Design and Development, Lilly Research Laboratories, Eli Lilly and Company, Indianapolis, IN 46221, United States*

Contents

Figure S1. Experimental setup for induction time measurement in the lab

Figure S2. The XRD patterns of glycine homopeptides before and after induction time measurement

Figure S3 Correlation of nucleation rate with the supersaturation of glycine homopeptides under different temperatures

Figure S4 Induction time of glycine homopeptides under different supersaturation levels at 278.15K and 283.15K. Solid lines represent the fit of the Poisson distribution

Table S1. The experimental conditions of the nucleation measurement of glycine, diglycine and triglycine

Table S2. The calculated nucleation kinetics for glycine homopeptides at 278.15 K and 283.15 K

Table S3. Values obtained from linear fit and calculated nucleation parameters for glycine homopeptides at 283.15K

Table S4. Values obtained from linear fit and calculated nucleation kinetics for glycine homopeptides at 278.15K

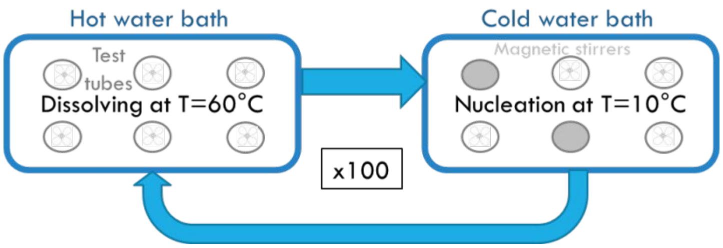

Figure S1. Experimental setup for induction time measurement in the lab

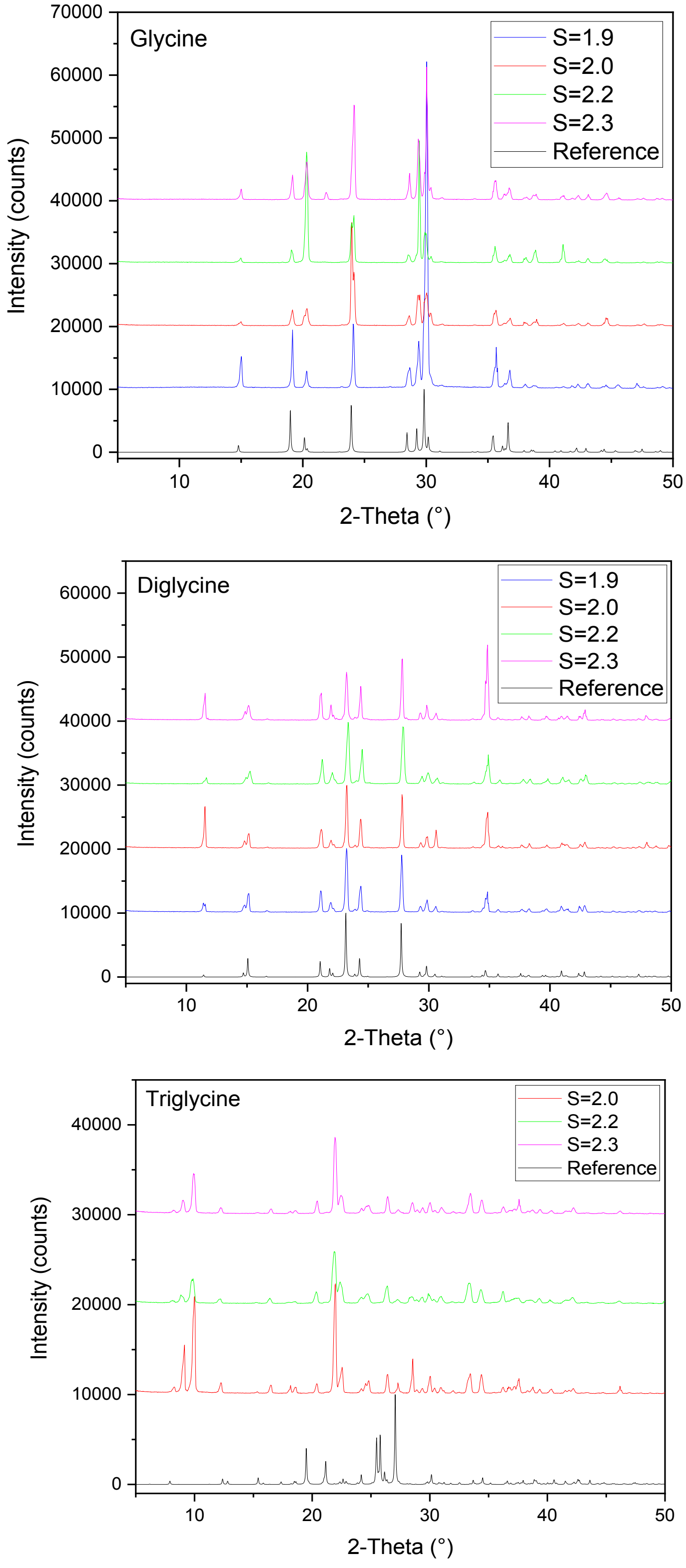

Figure S2. The XRD patterns of glycine homopeptides before and after induction time measurement. (For triglycine, the XRD patterns under different supersaturations all adopt the dihydrate's pattern. The reference indicates the XRD patterns of anhydrate.)

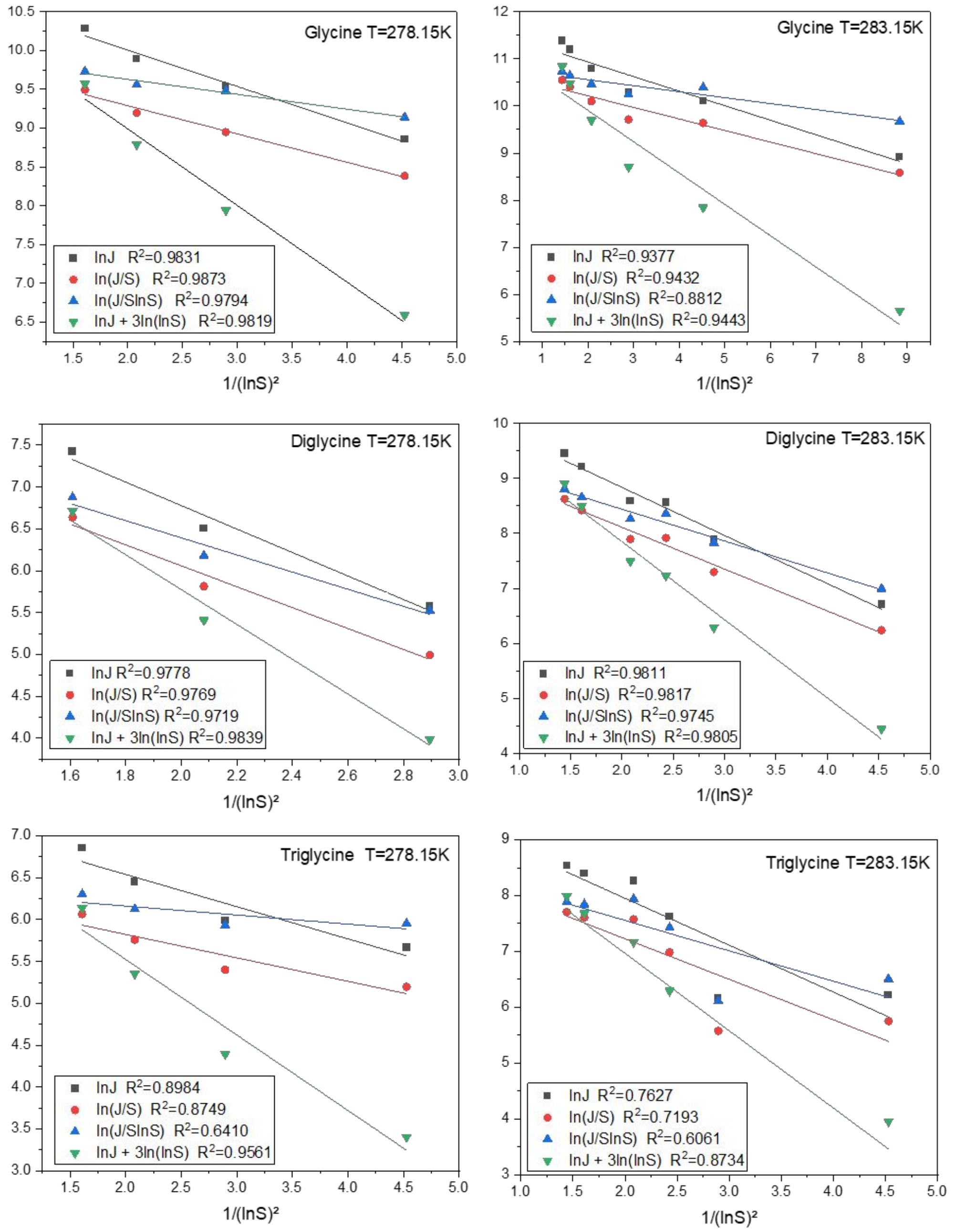

Figure S3 Correlation of nucleation rate with the supersaturation of glycine homopeptides under different temperatures

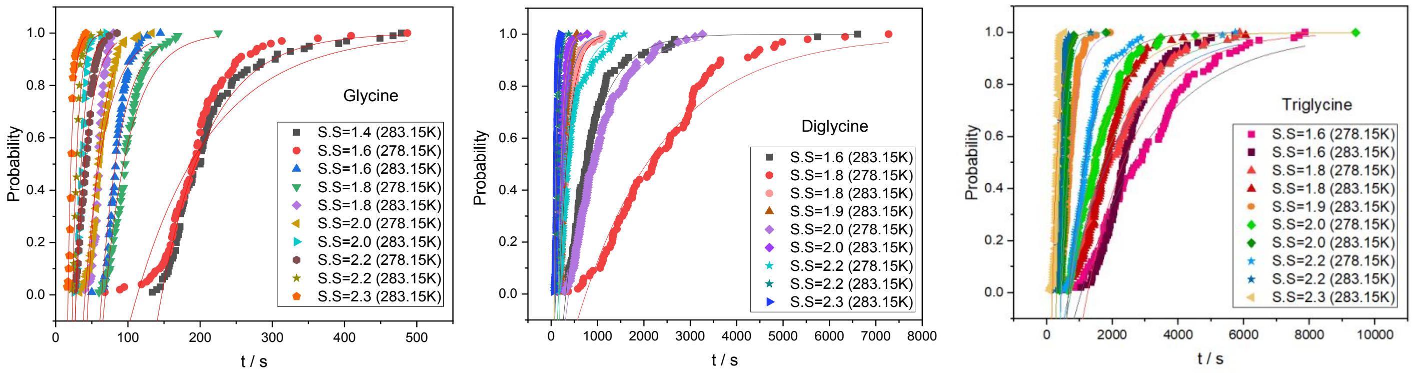

Figure S4. Induction time of glycine homopeptides under different supersaturation levels at 278.15K and 283.15K. Solid lines represent the fit of the Poisson distribution

Table S1. Properties of materials used in this work

Material	Molar Mass (g·mol-1)	CAS registry no.	Mass Fraction Purity	Source
Glycine	75.07	56-40-6	≥0.990	Sigma-Aldrich Company Ltd.
Diglycine	132.12	556-50-3	≥0.990	Sigma-Aldrich Company Ltd.
Triglycine	189.17	556-33-2	≥0.990	Sigma-Aldrich Company Ltd.
Tetraglycine	246.22	637-84-3	≥0.980	Sigma-Aldrich Company Ltd.
Pentaglycine	303.27	7093-67-6	≥0.980	Sigma-Aldrich Company Ltd.
Hexaglycine	360.32	3887-13-6	≥0.980	Sigma-Aldrich Company Ltd.

Table S2. The experimental conditions of the nucleation measurement of glycine, diglycine and triglycine

S	Glycine		Diglycine		Triglycine	
	278.15K	283.15K	278.15K	283.15K	278.15K	283.15K
1.4						
1.6						
1.8						
1.9						
2.0						
2.2						
2.3						

*The dark blue color shows conditions under 278.15K and the light blue color shows conditions under 283.15K.

Table S3. The calculated nucleation kinetics for glycine homopeptides at 278.15 K and 283.15 K

T/°C	T/K	V/cm³	S	R²	J /m⁻³ s⁻¹	t_g (s)	1/(lnS)²	lnJ	ln(J/S)	ln(J/SlnS)	lnJ + 3ln(lnS)
					Glycine						
10	283.15	2	1.4	0.9778	74700	146.8992	8.8329	8.9187	8.5822	9.6714	5.6509
10	283.15	2	1.6	0.9621	24600	67.8182	4.5269	10.1105	9.6405	10.3955	7.8455
10	283.15	2	1.8	0.8238	29690	46.4636	2.8944	10.2986	9.7108	10.2422	8.7044
10	283.15	2	2.0	0.9399	48670	28.1022	2.0814	10.7928	10.0997	10.4662	9.6933
10	283.15	2	2.2	0.9334	72760	23.9071	1.6086	11.1949	10.4065	10.6441	10.4819
10	283.15	2	2.3	0.8599	88130	17.1760	1.4415	11.3866	10.5537	10.7365	10.8381
5	278.15	2	1.6	0.9275	70150	135.3543	4.5269	8.8558	8.3858	9.1408	6.5908
5	278.15	2	1.8	0.9344	13845	68.7710	2.8944	9.5358	8.9479	9.4793	7.9415
5	278.15	2	2	0.9222	19720	42.4799	2.0814	9.8894	9.1962	9.5628	8.7898
5	278.15	2	2.2	0.9564	29225	28.3442	1.6086	10.2828	9.4943	9.7320	9.5697
					Diglycine						
10	283.15	2	1.6	0.9795	8200	319.1015	4.5269	6.7093	6.2393	6.9943	4.4443
10	283.15	2	1.8	0.9972	2655	196.0230	2.8944	7.8842	7.2964	7.8278	6.2900
10	283.15	2	1.9	0.9928	5215	196.9189	2.4273	8.5593	7.9174	8.3608	7.2291
10	283.15	2	2.0	0.9765	5385	151.3666	2.0814	8.5914	7.8982	8.2647	7.4918
10	283.15	2	2.2	0.9153	9990	95.8312	1.6086	9.2093	8.4209	8.6586	8.4963
10	283.15	2	2.3	0.89660	12795	72.8940	1.4415	9.4568	8.6239	8.8067	8.9083
5	278.15	2	1.8	0.95857	265.3485	748.1490	2.8944	5.5810	4.9936	5.5246	3.9869
5	278.15	2	2.0	0.99076	670	378.2064	2.0814	6.5073	5.8141	6.1806	5.4077
5	278.15	2	2.2	0.98397	1680	162.4202	1.6086	7.4265	6.6381	6.8758	6.7135
					Triglycine dihydrate						
10	283.15	1.5	1.6	0.8862	425.8047	1243.1838	4.5269	6.0540	5.5840	6.3390	3.7890
10	283.15	1.5	1.8	0.8953	480.2700	758.4529	2.8944	6.1743	5.5866	6.1180	4.5802

10	283.15	1.5	1.9	0.9672	2046.6667	448.3031	2.4273	7.6240	6.9821	7.4255	6.2938
10	283.15	1.5	2.0	0.8964	3893.3333	423.2787	2.0814	8.2670	7.5739	7.9404	7.1675
10	283.15	1.5	2.2	0.8897	4426.6667	295.5602	1.6086	8.3954	7.6069	7.8446	7.6824
10	283.15	1.5	2.3	0.8221	5106.6667	208.3258	1.4415	8.5383	7.7054	7.8882	7.9898
5	278.15	1.5	1.6	0.9770	289.4140	1047.4838	4.5269	5.6679	5.1979	5.9529	3.4028
5	278.15	1.5	1.8	0.9843	398.7593	686.0846	2.8944	5.9884	5.4057	5.9320	4.3942
5	278.15	1.5	2.0	0.9860	634.7140	674.9353	2.08141	6.4532	5.7600	6.1265	5.3536
5	278.15	1.5	2.2	0.9669	946.6667	665.6701	1.6086	6.8529	6.0645	6.3022	6.1399

Table S4. Values obtained from linear fit and calculated nucleation kinetics for glycine homopeptides at 278.15K

Molecular weight (g/mol)	Density (g/m^3)	v one molecule	S	A	B	γ/mJ·m^{-2}	r_c /nm	n_c	ΔGc /kJmol^{-1}
				Glycine					
75.07	1509550	8.26081E-29	1.6	57699.28	0.986	7.876	0.721	18.999	10.316
			1.8				0.576	9.714	6.596
			2.0				0.489	5.923	4.743
			2.2				0.430	4.024	3.666
				Diglycine					
132.12	1477200	1.48571E-28	1.8	20625.84	2.080	6.952	0.899	20.486	14.161
			2.0				0.762	12.492	10.183
			2.2				0.670	8.488	7.870
				Triglycine dihydrate					
225.17	1518350	2.46E-28	1.6	1523.552	0.902	3.690	1.007	17.381	9.437
			1.8				0.805	8.886	6.0340
			2.0				0.683	5.419	4.3390
			2.2				0.600	3.682	3.3534

Table S5. Values obtained from linear fit and calculated nucleation parameters for glycine homopeptides at 283.15K

Molecular weight (g/mol)	Density (g/m^3)	v one molecule	S	A	B	γ /mJ·m^{-2}	r_c /nm	n_c	ΔGc /kJmol^{-1}
				Glycine					
75.07	1509550	8.26E-29	1.4	76114.952	0.665	7.031	0.883	34.934	13.823
			1.6				0.632	12.817	7.084
			1.8				0.506	6.553	4.530
			2.0				0.429	3.996	3.257
			2.2				0.377	2.715	2.517
			2.3				0.357	2.303	2.256
				Diglycine					
132.12	1477200	1.48571E-28	1.6	43914.507	1.421	6.122	0.990	27.366	15.126
			1.8				0.792	13.991	9.671
			1.9				0.725	10.745	8.110
			2.0				0.671	8.532	6.955
			2.2				0.590	5.797	5.375
			2.3				0.559	4.917	4.816
				Triglycine dihydrate					
225.17	1518350	2.46344E-28	1.6	18839.293	1.4392	4.389	1.177	27.726	15.325
			1.8				0.941	14.176	9.798
			1.9				0.862	10.887	8.217
			2.0				0.798	8.644	7.046
			2.2				0.702	5.873	5.446
			2.3				0.664	4.982	4.880
